# Clinical Data Mega-Collection of Obesity and Obesity-Related Trials: Primary Inclusion Criteria from All Studies and Highlights of Clinical Efficacy Analysis of GLP-1 Drugs

**DOI:** 10.3390/jcm14030812

**Published:** 2025-01-26

**Authors:** Trung Tin Nguyen, David R. Elmaleh

**Affiliations:** 1 LizAI Inc., Newton, MA 02459, USA; 2 Department of Radiology, Massachusetts General Hospital and Harvard Medical School, Boston, MA 02114, USA

**Keywords:** obesity, overweight, clinical trials, data mining, data collection, data analysis, GLP-1, GI, semaglutide, Wegovy, tirzepatide, Zepbound, body mass index (BMI)

## Abstract

**Background/Objectives:** Obesity is heterogeneous and considered a chronic epidemic with significant un-met needs for management, treatment, and prevention. **Methods:** In this study, we used LizAI’s software TAITAN (alpha version) for the mega-collection and analysis of clinical data from 10,407 trials addressing obesity and obesity-related diseases and their associated publications, mainly on PubMed. **Results:** We report an intensive growth of clinical trials until the end of 2024 and highlight the use of the body mass index (BMI) as a critical criterion in clinical participant selection despite its limitations. The significant disparities in races, regions, and the sites of trials across all studies have not been addressed, posing the possibility of research in the far future on the applications of precision medicine in weight management. In the latter parts of this paper, we analyze and discuss the clinical efficacy, mainly focusing on the primary endpoints and benchmarks of the recently FDA-approved once-weekly injectable glucagon-like peptide-1 receptor agonist (GLP-1 RA) drugs, including semaglutide and tirzepatide. Both drugs have functioned comparably when considering the 5% weight loss FDA threshold. Tirzepatide outperforms semaglutide and impacts fewer participants as the weight loss level increases from 5 to 20% and has greater effects in different populations, especially in people with type 2 diabetes (T2D). **Conclusions:** We would, however, like to highlight that (i) the weight loss level should be dependent on the clinically relevant needs of patients, and faster and greater weight loss might not be a win, and (ii) the clinical benefits, safety, and quality of life of patients should be carefully assessed when the weight loss is significant in a short period. In our search, we found that the specificities and impacts of weight loss therapies on organs like the kidneys and heart, different muscle types, bones, and fat accumulation in different parts of body were not investigated or disclosed during the clinical study period and longer term monitoring. In light of scientific needs and remarkable public interest in weight loss, our report provides findings on the buzz around losing weight in clinical trials, and our TAITAN software continues to collect data in real time and enrich its knowledge for future updates.

## 1. Introduction

Obesity indicates the abnormal or excessive accumulation of fat in people, which poses health risks, and was recognized as a chronic disease in 1997 by the World Health Organization (WHO) [1,2]. The overweight and obese population has hit nearly half of the total global human population since 2022, including almost 3.4 billion adults (18 years and older) and 550 million children [3]. The impacted population continues to grow, and this trend is a significant public health concern due to the effects on the quality of life and the health-associated risks, mainly heart disease, diabetes, and cancers [4,5,6]. Thus, major public health organizations, including the WHO, the American Medical Association (AMA), and the Centers for Disease Control and Prevention (CDC), have considered obesity a global epidemic and called for immediate management, treatment, and prevention strategies [7,8,9].

Obesity is a complex and heterogeneous disease that is influenced by a variety of biological, behavioral, and cultural factors [10,11]. Considerable geographical, national, racial, ethnic, and gender disparities which affect the prevalence of overweight and obesity have been reported [12,13]. Variations in socioeconomic and ecological factors drive the disparities [8,13,14,15]. Most importantly, a genetic predisposition exacerbates the susceptibility of some populations, such as Black or African American people and Middle Easterners, causing significant racial and ethnic disparities in obesity [16,17]. These systematic differences emphasize that there is no “one solution fits all” in obesity, and tailored medical strategies—also called precision medicine—and management policies are urgently needed to address these disparities [18].

The primary goal of obesity management and weight-related risk mitigation is weight loss, whose targets should depend on the patient’s initial body weight and obesity-related comorbidities [19,20]. A five percent or more weight loss is associated with improved blood pressure and glycosylated hemoglobin (HbA1c) levels. In comparison, higher weight loss of up to ten percent is associated with enhanced lipidomics and the prevention of type 2 diabetes (T2D) [21]. Weight loss in the range of 10–15% and greater has resulted in, specifically, the remission of T2D (not fully cured), improvement in patients with heart failure, and reduced cardiovascular mortality [22,23]. In general, the clinical approaches for weight loss include lifestyle interventions, behavioral and psychological therapy, pharmacotherapy, and bariatric surgery, among which pharmacotherapy is more advanced, more available, and more efficacious with at least five percent weight loss than the others, thereby rendering it an attractive option to bridge this treatment gap [24].

In light of the scientific focus and social needs, we report here the clinical development progress and disparities, which use clinical criteria, such as the BMI and other clinical indicators, in finding medications for obesity management, treatment, and prevention. We employed TAITAN software for data mega-collection and provide an independent analysis of 10,407 clinical trials associated with overweight and obesity reported on clinicaltrials.gov and their related publications since the 1980s. We then highlight and discuss clinical trial data and clinical considerations, especially the primary outcomes, indications, and adverse effects of once-weekly subcutaneous injectables of semaglutide and tirzepatide as medications for weight loss.

## 2. Method for Data Collection and Analysis

At LizAI Inc., we have built an innovative software called TAITAN (alpha version) that is capable of collecting, structuring, and analyzing data from many sources, with advantages in terms of specificity and accuracy for multiple feature searches beyond the currently available data relationship based on large language models. We collected published information from 517,553 studies on clinicaltrials.gov and their associated publications, mainly on PubMed, up until the time of this report, and TAITAN continues to collect data in real time to enrich its knowledge in the selected fields.

In TAITAN, we then extracted information based on the list of pre-defined obesity and obesity-related conditions (Table S1: List of obesity and obesity-related disease conditions used for the search in this study), from which we found 10,407 clinical studies focusing on obesity and related diseases. The software specifically extracted the information and data in text, tables, and figures and all related information from these 10,407 trials. The search inputs include the following categories: active ingredients, doses, administration requirements, treatment designs, trial information, the sites of trials, primary inclusion criteria for participants, exclusion criteria for participants, participants’ diversity, clinical primary outcomes, clinical secondary outcomes, clinical endpoints and benchmarks, US Food and Drug Administration (FDA)-approved indications, and adverse events (both serious and other). All extracted data were automatically structured, coded into datasets as pre-defined categories, and analyzed by the software.

This report provides insights into the clinical development progress of weight management medications and their clinical efficacy. In the sections discussing glucagon-like peptide-1 receptor agonist (GLP-1 RA) drugs, we pull data from different trials which present the possible therapeutic interpretation in a large population and discuss the medicines’ indications and adverse effects. We did not perform additional statistical analysis for the data from different trials in this report, as each trial had its own design and purposes. 

## 3. Results and Discussion

### 3.1. The Body Mass Index (BMI) as an Indication of Obesity

The body mass index (BMI) is a measure of weight relative to height (Equation (1)), was introduced in 1972, and has undergone minimal changes since then [25,26].

(1) $$\mathrm{BMI}=\frac{Weight(kg)}{{\left[Height\left(m\right)\right]}^{2}}$$

Objectively, individual variations exist, and the BMI alone is insufficient to classify a person as overweight or obese and does not represent the body fat mass and its relationship to mortality and various morbidities. Additionally, numerous comorbidities, lifestyle issues, gender, ethnicity, genetic factors, and the expected accumulation of fat with aging significantly affect the interpretation of BMI data. Consequentially, other considerations, methods, and measurements are utilized when considering weight-related medications, such as waist-to-hip ratios, the skin impedance, dietary cycling, dual X-ray absorptiometry, and other weight-related diseases [26,27].

Despite the limitations of using the BMI, it is still the most widely used indicator of obesity and one of the standards for clinical decisions. The BMI values and classifications are listed in Table 1. The Asian group is heterogenous and has lower recommended BMI cutoffs for obesity. Subgroups of the Asian population are further divided into regions whose BMI classifications are assessed differently in clinical practice, such as in Japan, Korea, and China [28].

### 3.2. Numerous Clinical Trials for Obesity and Obesity-Related Conditions, the Disparities in Regions and Races, and the Popularity of the BMI

**Figure 1:**
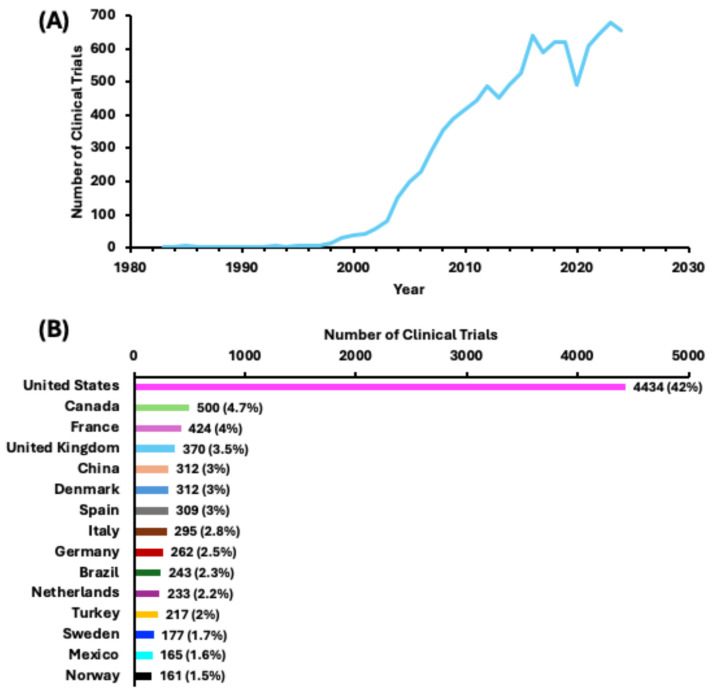
(**A**) The growth of obesity and obesity-related clinical trials from 1983 to 2024, totaling 10,407; (**B**) the top 15 countries with the highest number of obesity and obesity-related clinical studies among the 10,407 trials.

The rising prevalence of obesity and overweight has led to their recognition as diseases [5,29]. Consequently, the number of clinical trial registrations addressing obesity in the FDA database has increased exponentially since 1983. The software collected published information from 10,407 clinical studies, focusing on obesity and related diseases and summarized the numbers of trials in Figure 1A. The analysis included all trials, including those that were recruiting, ongoing, active, completed, withdrawn, terminated, approved for marketing status, and more.

The locations, including the countries and clinical sites, of each trial are reported in Figure 1B and Table S2. The U.S. hosts the highest number of trials to date, accounting for 42% (4434) of studies (Figure 1B), indicating the remarkable market size in urgent need of weight management. Interestingly, the top 15 countries for obesity and overweight studies are in North America and Europe, while the top countries with the highest percentage of the obese population according to the Global Obesity Observatory (https://data.worldobesity.org (accessed on 15 December 2024)) [28], especially the Middle Eastern countries—such as Egypt, Qatar, Saudi Arabia, and Kuwait—have not yet researched this extensively. China is the only Asian country in this top 15 list, with 312 studies. Despite the increase in the search for obesity medications, this field still holds significant potential and continues to require innovations in both scientific and marketing strategies which will address the disparities in regions and races.

**Figure 2:**
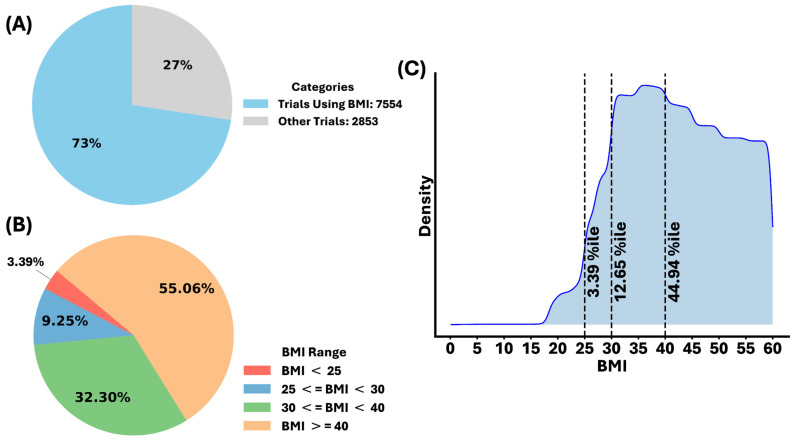
(**A**) Distribution of trials requiring BMI in inclusion criteria among 10,407 clinical trials (note: strictly considered for inclusion criteria—100% of studies used BMI as clinical factor, either as primary or secondary measurement); (**B**) distribution of BMI ranges across 7554 trials requiring BMI in inclusion criteria; (**C**) density of BMI ranges across 7554 trials requiring BMI in inclusion criteria.

Despite the enormous obese and overweight population worldwide, the BMI is still one of the major factors for selecting candidates in clinical studies. Among the 10,407 trials, there were nearly 7600 that utilized the BMI as a strict inclusion criterion, accounting for ~75% of all studies (Figure 2A). The inclusion of other studies that used the BMI as a secondary consideration drove the total use of the BMI to 100% of all trials. It is important to note that the BMI was also considered as the only inclusion criterion in some trials. There are relevant clinical limitations in using the BMI for obesity and overweight when it comes to the body fat mass [26], but it is still challenging to completely replace the BMI with other factors. However, in addition to the BMI, additional tests and measurements, for example, waist-to-hip ratios, the skin impedance, dietary cycling, and dual X-ray absorptiometry, have been added to strengthen clinical investigations [26,30].

Notably, our system found that the population with a BMI > 30 kg/m^2^ accounted for more than 85% of the total participants in 7554 trials, which is a dramatic number and a relevant representation for the obese population (Figure 2B). Additional populations with a different BMI range versus their density are presented in Figure 2C, showing that the studies covered a broad range of BMI values.

**Figure 3:**
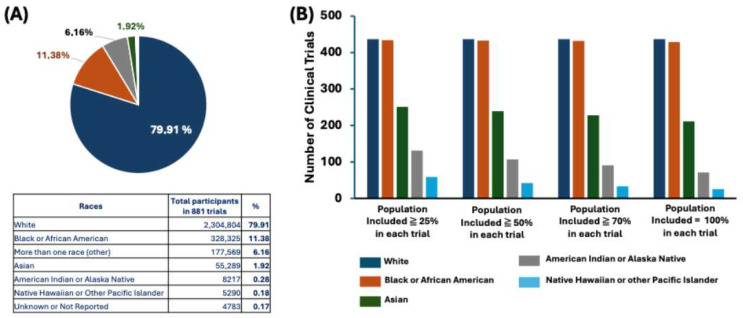
(**A**) Distribution of races among 881 obesity and obesity-related clinical trials which had published results on clinicaltrials.gov; (**B**) number of clinical trials with different inclusion percentage of each population.

As the majority of the clinical trials were conducted in the US, whose population is diverse and highly impacted by obesity [8], we used our data collection system to analyze the distribution of race in the population in nearly 900 trials. The participation of White volunteers was overwhelmingly higher than that of any others, comprising 80% of the whole population (Figure 3A). The associated table in Figure 3A provides detailed numbers and percentages for all races of interest, including White, Black or African American, more than one race (other), Asian, American Indian or Alaska Native, native Hawaiian or other Pacific Islander, and unknown or not reported. The total Black population who had joined trials related to obesity was very limited, only nearly 11%. The CDC has reported that the prevalence of obesity and even severe obesity has been the highest among non-Hispanic Black adults compared with other races and Hispanic origin groups, and the overall obesity rate in adult women is higher than that in adult men [31,32].

Additionally, the standard measurement, in particular the BMI, of the Asian obese population is different from that of the general population (as indicated in Table 1) [33,34]. The efficacious outcomes of the same treatment methods might thus be varied in the Asian group, and it is challenging to interpret and compare the effects of therapeutics among races when only including a small portion of the minority.

Although the total population shows significant disparity in race inclusion, Figure 3B indicates that there have been a number of trials in which a specific population was prioritized and included. This might fulfill the clinical criteria for drug approval consideration, but the clinical factors, especially the therapeutic efficiency and adverse effects impacting a larger population with overweight and obesity, still need to be addressed. As a result, the investigation and applications of precision medicine will still take a long time to become reality under the current approach. Additional clinical study designs which exclusively recruit a sufficiently large number of a minority population, such as the Black population, and are run in specific regions, such as Middle Eastern countries, will resolve the disparities in regions and races. A clinical trial offers safe and effective indications to doctors and patients; however, it is lengthy and does not meet urgent medical needs. The current artificial intelligence (AI) approach might enhance data collection and analysis, accelerate the trials, and help predict the outcomes in different regions and races more efficiently. The AI approach holds promise, although it still requires remarkable technological advancements in data collection, clinical prediction, and clinical validation.

### 3.3. The Search for Medications for Weight Management

There have been many clinical trials advancing the medications for obesity management and treatment. Only six weight loss medications have been approved by the FDA for long-term chronic weight management up to today, including orlistat (a lipase inhibitor), phentermine–topiramate, naltrexone–bupropion (neurotransmitter agonists and re-uptake inhibitors), liraglutide, semaglutide (GLP-1 RA) [35], and most recently, tirzepatide (a dual gastric-inhibitory [GI] polypeptide analog and GLP-1 RA) [36].

As a result of our investigation, Table 2 presents the top ten (10) active compounds that have been most studied in trials for weight management. Interestingly, many of these were approved for the management of T2D before they were evaluated for obesity and overweight [35]. The primary focus in recent years has been a once-weekly injection of GLP-1 RA compounds, centering around two main competitors—Novo Nordisk developed semaglutide, branded as Wegowy, and Eli Lilly own the dual GI analog and GLP-1 RA tirzepatide—who have conducted intensive investigations and have the highest number of clinical trials. We will discuss the approved indications and clinical outcomes of these two compounds in the next sections.

It is worth noting that the FDA’s guidance does not explicitly discuss weight loss or the maintenance of weight loss as indications in using medications for weight management, both of which, however, should be demonstrated over the course of at least 1 year before a medicine can be considered adequate. The weight loss should be equal to or greater than 5% compared to the baseline. Additionally, a reasonable phase 3 design for a weight management product should include a total of approximately 3000 subjects who are randomized to placebo and active doses for 1 year of treatment in order to investigate the efficacy and safety sufficiently [37]. Consequently, the search for weight management medications is lengthy and challenging, and the number of approved medicines clearly does not meet the current market demands.

### 3.4. FDA-Approved Once-Weekly Injection of GLP-1 Compounds: Semaglutide and Tirzepatide

Semaglutide was initially approved in 2017 by the FDA, and then in 2018 by the European Medicines Agency (EMA), as a once-weekly injectable treatment at doses of up to 1.0 mg for managing T2D. The once-weekly injectable semaglutide 2.4 mg was approved by the FDA (US), Health Canada (Canada), and the Medicines and Healthcare Products Regulatory Agency (UK) in 2021, and by the EMA (Europe) in 2022, as an adjunct treatment for weight management, in conjunction with dieting and exercise [38]. A once-weekly injection of up to 15 mg tirzepatide was newly approved by the FDA in late 2023 for weight management and also as an adjunct to a reduced calorie diet and increased physical activity [36].

The FDA indications for both drugs are quoted in Table 3; these share similar criteria to patient selection, especially regarding the BMI and the presence of at least one weight-related comorbid condition. Some comorbidities are given as examples in both indications, including hypertension, type 2 diabetes, dyslipidemia, obstructive sleep apnea, or cardiovascular disease. The indication of weight-related comorbid conditions is still very vague, considering that there are 200 and even more clinically relevant comorbidities associated with weight [39,40].

Studies have shown the benefits of weight management in weight-related risk mitigation [41,42]. The effects of weight management medicines like semaglutide and tirzepatide on obese and overweight patients with pre-existing conditions should be investigated case by case. We analyzed our database, and the most studied weight-related comorbidities are summarized in Table 4. While the benefits of weight loss might be more favorable in a risk–benefit consideration, more apparent approved indications that should not be used beyond these conditions would provide better clarity to healthcare providers.

Furthermore, the use of semaglutide has been explored in some studies for many other health conditions beyond weight-related diseases, such as Alzheimer’s disease, Parkinson’s disease, and addiction, as of the end of 2024 [43]. While their clinical benefits are still being investigated, the actual usage of both semaglutide and tirzepatide should be clearly guided and regulated.

### 3.5. The Highlights of the Clinical Efficacy Analysis of Phase 3 Trials of Semaglutide and Tirzepatide

#### 3.5.1. The Selection of Phase 3 Trials in This Report

For this section, we selected phase 3 trials of semaglutide and tirzepatide and further analyzed the clinical outcomes by pulling all the published data together. It was noted that each clinical trial had its own design, setting, and goals, and comparing some specific outcomes might thus have led to data misinterpretation and mis-conclusions. Based on the FDA-approved indications regarding the vague inclusion of weight-related comorbidities for clinically qualified adults, we report and compare the primary outcomes in the later sections, focusing on the most crucial feature—the weight loss percentage compared to the placebo—and some outstanding outcomes, including the percentage of participants reaching a weight loss equal to or higher than 5, 10, 15, and 20 percent, and eventually the BMI. Additional statistical analysis for the data from different trials was not performed. Table 5 includes the trials of interest for our report.

#### 3.5.2. Primary Efficacy Endpoints and Benchmarks

Two (2) significant factors, including (i) the mean percent loss and (ii) the proportion of subjects with at least a 5 percent loss of their baseline body weight in treatment groups, were considered the primary efficacy endpoints of an investigated therapy for weight management when they were significantly different to the placebo-treated group [37]. An investigational weight management therapy can be considered efficacious if, after one (1) year of treatment, either of the following occurs:

- There is at least 5 percent mean weight loss in the investigational therapy-treated group versus placebo-treated groups, and the difference is statistically significant.
- At least 35 percent of participants lose 5 percent or greater of their baseline body weight in the investigational therapy-treated group, which should be approximately double the proportion in the placebo-treated group, and the difference between groups is statistically significant.

Additionally, other changes in participants’ clinical conditions and prevalent weight-related comorbidities should be factored into the efficacy assessment of investigational weight management therapies [37].

**Figure 4:**
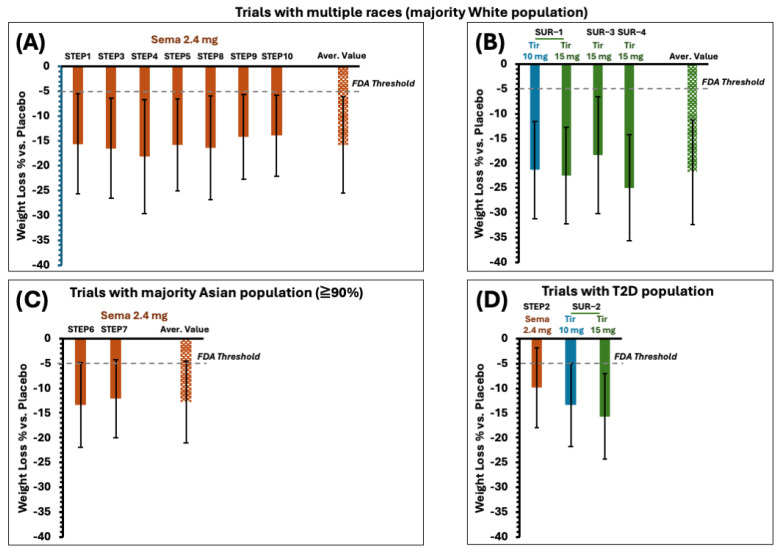
A summary of the mean weight loss percentages in the weight management drug-treated group versus the placebo-treated group in the trials with multiple races in which the White population was the majority: (**A**) STEP trials with once-weekly injectable semaglutide 2.4 mg (Sema 2.4 mg); (**B**) SURMOUNT (SUR) trials with once-weekly injectable tirzepatide (Tir). (**C**) Trials with a majority Asian population who received once-weekly injectable Sema 2.4 mg. (**D**) Trials with a population with type 2 diabetes (T2D). The dashed line indicates the FDA threshold for the 5 percent mean weight loss in the investigational therapy-treated group versus the placebo-treated groups, and the difference is statistically significant. All data were recorded at week 44 or later after the treatment.

Figure 4 summarizes the primary endpoint—the weight loss percentage—across all STEP trials and SURMOUNT trials that have already published their data. Despite the differences in the design and goals, the mean weight loss percentages were comparable in different trials for both semaglutide and tirzepatide, in which the majority of participants belonged to the White population (Figure 4A,B). In the trials with a greater proportion of the Asian population (Figure 4C), the overall weight loss efficacy of semaglutide remained like that of other trials, with a minor decrease. At the same time, tirzepatide has not been investigated in similar trial designs yet. Previous studies have confirmed that overweight and obese patients with T2D often respond less favorably to weight management products compared with patients without diabetes. The underlying reasons are still unclear, and additional programs are needed to understand the relationships between weight management treatment outcomes for patients with and without T2D. In this case, tirzepatide showed advantageous outcomes compared to semaglutide’s trials (Figure 4D). The general weight loss performance of tirzepatide was relatively higher than semaglutide by 5%; both drugs, however, showed broad standard deviations in their outcomes, which might lead to comparable therapeutic impacts in an application for a large population. This feature will be further discussed in conjunction with the proportion of participants with different levels of weight loss.

**Figure 5:**
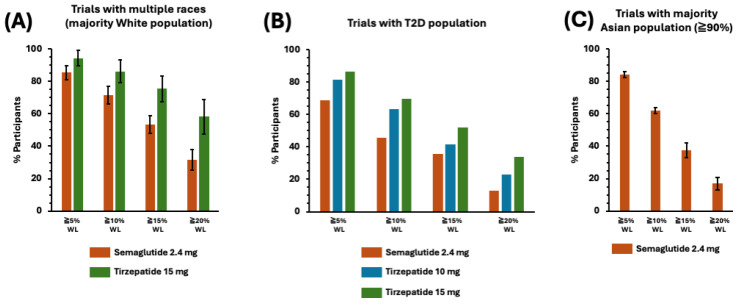
A summary of the participant proportion with a weight loss (WL) percentage equal to or higher than 5, 10, 15, and 20% in (**A**) trials with multiple races in which the White population was the majority; (**B**) trials with a population with type 2 diabetes (T2D); and (**C**) trials with a majority Asian population. All data were recorded at week 44 or later after the treatment and presented as an average value for the relevant trials listed in Table 5.

The once-weekly injectable tirzepatide performed better than semaglutide when considering the proportion of patients with and without T2D who benefited from the 5 percent or more significant weight loss, and the efficacy gap became larger and more favorable for tirzepatide as the weight loss percentage increased to 20% (Figure 5A,B), which would drive more significant impacts on weight management in a large population setting. It is worth noting that weight loss of 20% or higher still occurred in an average of ~35% of participants treated with semaglutide (Figure 5A), which is an FDA threshold and significant enough for an effective weight management drug at the 20% or greater weight loss level. Figure 5C highlights a sharp decrease in the efficacy of semaglutide in the Asian population when the weight loss level increases to higher than 15%.

**Figure 6:**
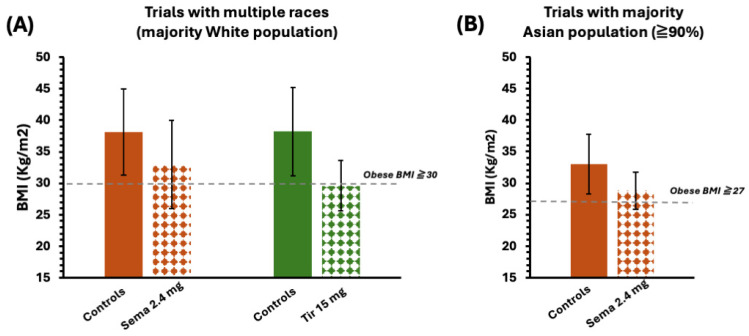
A summary of the BMI (**A**) in trials with multiple races in which the White population was the majority and (**B**) in trials with a majority Asian population. All data were recorded at week 44 or later after the treatment and presented as an average value for the relevant trials listed in Table 5.

We additionally summarize the BMI of patients after at least 44 weeks of treatment with semaglutide and tirzepatide in Figure 6. As we discussed above, this measurement might not be relevant for the body fat portion, but we could still use it for general information. The average BMI of patients did not reach the healthy level and had broad standard deviations in all trials, again showing the heterogeneity of obesity.

Weight loss should depend on patients’ clinically relevant needs, and faster and greater weight loss should not be an impactful feature. In turn, patients’ clinical benefits, safety, and quality of life should be carefully assessed when the weight loss is significant in a short period.

#### 3.5.3. Adverse Events

Semaglutide and tirzepatide have been reported to be safe and have not caused discontinuation due to death in their trials. Adverse effects, however, were reported at a high rate in all trials. Overall, clinical adverse events were reported in two big groups, including the serious adverse events which might be life-threatening and the other (non-serious) adverse events which were reported by the participants and/or clinicians. Both groups are important for consideration when prescribing the medicines to patients and might play a critical role in adherence and the final efficacy of the treatment. The categories of adverse events and the proportion of participants impacted by each adverse event are reported in Table 6.

Tirzepatide again showed some slight advantages over semaglutide, as tirzepatide impacted fewer participants in terms of both serious and other adverse events, considering a similar treatment period. This is surprising as tirzepatide was dosed at a much higher amount in the active group, up to 15 mg weekly, compared to that of semaglutide at 2.4 mg. These data still need to mature as the total number of trials and participants investigated with tirzepatide are less than those of semaglutide. These slight advantages will additionally need to be proved in additional studies with larger numbers of participants, especially in anticipation of the official data release from the SURMOUNT-5 trial (NCT05822830).

## 4. Conclusions

The ultimate utility of and interest of the public in weight loss and the associated side effects prompted us to review the buzz around losing weight in clinical trials. In this report, we used LizAI’s software TAITAN for the mega-collection and analysis of clinical data from clinicaltrials.gov and their associated publications, mainly on PubMed. Based on the extracted information from 10,407 clinical studies related to obesity and obesity-related conditions, we provided an overview of the intensive growth and needs in the search for weight management therapeutics until the end of 2024. Despite clinical limitations, the BMI is still a critical inclusion feature for participant selection in all trials for obesity and related diseases. Obesity is heterogeneous and considered a chronic epidemic, and the disparities in races across all trials were significant. The application of precision medicine in weight management would thus be unrealistic with the current approach/investigations and requires innovations.

We highlighted the clinical efficacy, mainly focusing on the primary endpoints and benchmarks of the recently FDA-approved once-weekly injectable GLP-1 RA drugs, including semaglutide and tirzepatide. The overall data from relevant trials show comparable outcomes when considering the 5% weight loss threshold for both semaglutide and tripeptide. Tirzepatide outperforms semaglutide as the weight loss level increases in different populations, especially with T2D. Additionally, our findings fully aligned with previous reports that tirzepatide has impacted fewer participants with adverse events [44]. We would, however, like to highlight that (i) the weight loss level should be dependent on the clinically relevant needs of patients, and faster and greater weight loss might not be a win, and (ii) the clinical benefits, safety, and quality of life of patients should be carefully assessed when the weight loss is significant in a short period.

Additionally, weight loss and management medications benefit the quality of life and mitigate weight-related health risks. However, regarding the specificities and consequences of weight loss therapies when it comes to organs, different muscle types, bones, fat accumulation in different parts of body, and more, these factors have been investigated and reported preclinically, which has shown some concerns regarding, for example, lean mass loss [45,46] and negative impacts on the kidneys and heart [47]. In our search and analysis, the current publications on clinical outcomes did not disclose and/or include the impacts of weight loss drugs on the abovementioned factors, especially the impacts of GLP-1 drugs and GI/GLP-1 drugs, during the study period and longer term monitoring.

We conclude that the weight management market is vast, and only six (6) medications have been approved, of which two (2) work as weekly injections and are not yet considered as options to fully treat obesity. This space has attracted significant attention and investment, which will continue to grow. The excitement around artificial intelligence and its applications in drug development and clinical trial acceleration will hopefully advance the search for additional obesity medications. We believe that the BMI is a significant factor in these studies. However, the studies should include at least one more measure in combination, such as a fat and skeletal muscle measurement at the start and end of the trial. The loss of skeletal muscle replacement with age may be a critical measure that contributes to high weight loss.

This work reported data collection and a fully independent analysis of the data from manufacturers of FDA-approved drugs. The publicly available data from clinicaltrials.gov and PubMed do not include the raw clinical data of each volunteer. This lack of full data disclosure from clinical trials, which allows for independent analysis and detailed data interpretation, represents the main limitation in clinical research and also in this article.

## Figures and Tables

**Table 1:** Values and classifications of body mass index (BMI) *.

General Population Classifications	
BMI (kg/m^2^)	Classifications
BMI < 16.5	Severely underweight
16.5 ≤ BMI < 18.5	Underweight
18.5 ≤ BMI < 24.9	Normal weight
25.0 ≤ BMI < 29.9	Overweight
30.0 ≤ BMI	Obesity
30.0 ≤ BMI < 34.9	Obesity class I
35.0 ≤ BMI < 39.9	Obesity class II
40.0 ≤ BMI	Obesity class III
**Asians and Asian Americans**	
23.0 ≤ BMI < 26.9	Overweight
27 ≤ BMI	Obesity
Japan and Korea **	
23.0 ≤ BMI < 24.9	Overweight
25 ≤ BMI	Obesity
**China**	
24.0 ≤ BMI < 27.9	Overweight
28 ≤ BMI	Obesity

* In the pediatric population, a BMI below the 5th percentile is considered underweight, while a BMI above the 95th percentile is considered obese. ** Some Asian countries, such as Japan and Korea, have different cutoffs for obesity, which use a BMI greater than 25 kg/m^2^ for obesity [28].

**Table 2:** Top ten (10) active ingredients that have been investigated in obesity and obesity-related clinical trials, ranked by the total number of trials.

Drug	Number of Trials
semaglutide	110
liraglutide	98
metformin	85
tirzepatide	44
topiramate	35
orlistat	34
exenatide	32
phentermine	30
bupropion	28
sibutramine	27

**Table 3:** FDA-approved indications of semaglutide and tirzepatide.

**Semaglutide** Branded as WEGOVY^®^ Owned by Novo Nordisk	Approved for chronic weight management in 2021 by the FDA. *Indications*: WEGOVY^®^ is indicated as an adjunct to a reduced calorie diet and increased physical activity for chronic weight management in the following: Adults with an initial body mass index (BMI) of 30 kg/m^2^ or greater (obesity) or 27 kg/m^2^ or greater (overweight) in the presence of at least one weight-related comorbid condition (e.g., hypertension, type 2 diabetes mellitus, or dyslipidemia). Pediatric patients aged 12 years and older with an initial BMI at the 95th percentile or greater, standardized for age and sex (obesity).
**Tirzepatide** Branded as ZEPBOUND^®^ Owned by Eli Lilly	Approved for chronic weight management in 2023 by the FDA. *Indications*: ZEPBOUND^®^ is indicated as an adjunct to a reduced calorie diet and increased physical activity for chronic weight management in adults with an initial body mass index (BMI) of the following: 30 kg/m^2^ or greater (obesity) or 27 kg/m^2^ or greater (overweight) in the presence of at least one weight-related comorbid condition (e.g., hypertension, dyslipidemia, type 2 diabetes mellitus, obstructive sleep apnea, or cardiovascular disease).

**Table 4:** Weight-related comorbidities that have been considered and/or investigated in participants in trials of semaglutide and tirzepatide.

Weight-Related Comorbidities	Counts in Clinical Trials
Hypertension	41
Metabolic Syndrome	41
*Dyslipidemia/Dyslipidaemia (38 counts)*	
Cardiovascular and Heart Disease	36
*Cardiovascular Disease (22 counts)*	
*Cardiac Conditions (10 counts)*	
*Coronary Artery Disease (1 count)*	
*Congestive Heart Failure (1 count)*	
*Myocardial Infarction (1 count)*	
*Peripheral Arterial Disease (1 count)*	
Sleep Apnea	26
Diabetes	21
*Type 2 Diabetes Mellitus (T2D) (16 counts)*	
*Insulin Resistance (2 counts)*	
*Prediabetes (2 counts)*	
*Type 1 Diabetes (1 count)*	
Polycystic Ovary Syndrome (PCOS)	8
Kidney Disease	2
*Chronic Renal Disease (1 count)*	
*Albuminuria (1 count)*	
Stroke	1
Non-alcoholic Fatty Liver Disease (NAFLD)	1

**Table 5:** Phase 3 clinical trials of semaglutide and tirzepatide for obesity and obesity-related comorbidities which had published data until the end of 2024 and whose data were used for analysis in this report.

Trial No.	Trial Name	Title	Notes
Semaglutide: Trials			
NCT03548935	STEP 1	Research Study Investigating How Well Semaglutide Works in People Suffering From Overweight or Obesity	Largest population: White, ~83%
NCT03552757	STEP 2	Research Study Investigating How Well Semaglutide Works in People With Type 2 Diabetes Suffering From Overweight or Obesity	Largest population: White, ~60%
NCT03611582	STEP 3	Research Study to Look at How Well Semaglutide is at Lowering Weight When Taken Together With an Intensive Lifestyle Program	Largest population: White, ~76%
NCT03548987	STEP 4	Research Study Investigating How Well Semaglutide Works in People Suffering From Overweight or Obesity	Largest population: White, ~84%
NCT03693430	STEP 5	Two-year Research Study Investigating How Well Semaglutide Works in People Suffering From Overweight or Obesity	Largest population: White, ~93%
NCT03811574	STEP 6	Research Study Investigating How Well Semaglutide Works in People Living With Overweight or Obesity	Largest population: Asian, 100%
NCT04251156	STEP 7	Research Study of How Well Semaglutide Works in People Living With Overweight or Obesity	Largest population: Asian, 90%
NCT04074161	STEP 8	Research Study to Investigate How Well Semaglutide Works Compared to Liraglutide in People Living With Overweight or Obesity	Largest population: White, 75%
NCT05064735	STEP 9	Research Study Looking at How Well Semaglutide Works in People Suffering From Obesity and Knee Osteoarthritis	Largest population: White, 62%
NCT05040971	STEP 10	Research Study Looking at How Well Semaglutide Works in People Living With Obesity and Prediabetes	Largest population: White, 90%
**Tirzepatide: Trials**			
NCT04184622	SURMONT-1 (SUR-1)	A Study of Tirzepatide (LY3298176) in Participants With Obesity or Overweight	Largest population: White, ~71%
NCT04657003	SURMONT-2 (SUR-2)	A Study of Tirzepatide (LY3298176) in Participants With Type 2 Diabetes Who Have Obesity or Are Overweight	Largest population: White, ~80%
NCT04657016	SURMONT-3 (SUR-3)	A Study of Tirzepatide (LY3298176) In Participants After A Lifestyle Weight Loss Program	Largest population: White, ~86%
NCT04660643	SURMONT-4 (SUR-4)	A Study of Tirzepatide (LY3298176) in Participants With Obesity or Overweight for the Maintenance of Weight Loss	Largest population: White, ~80%

**Table 6:** Summary of adverse events reported when patients were treated with once-weekly injectable semaglutide 2.4 mg and tirzepatide at different doses. The serious adverse events might be life-threatening, while the other (non-serious) adverse events were reported by the participants and/or clinicians. All data were recorded at week 44 or later after the treatment and presented as an average value for the relevant trials listed in Table 5.

Adverse Event Categories	Other Adverse Events				Serious Adverse Events			
	Semaglutide		Tirzepatide		Semaglutide		Tirzepatide	
	% Reported	SD	% Reported	SD	% Reported	SD	% Reported	SD
Gastrointestinal disorders	14.70	12.34	12.90	9.15	0.19	0.26	0.09	0.14
General disorders	7.00	2.74	5.66	2.96	0.18	0.25	0.06	0.11
Infections and infestations	10.33	6.97	8.88	6.33	0.16	0.24	0.13	0.20
Injury, poisoning, and procedural complications	6.58	0.0	-	-	0.14	0.23	0.08	0.12
Metabolism and nutrition disorders	8.18	3.12	7.32	3.90	0.12	0.14	0.24	0.14
Musculoskeletal and connective tissue disorders	6.51	2.74	4.18	2.46	0.16	0.22	0.08	0.13
Nervous system disorders	8.59	4.57	5.47	2.02	0.12	0.17	0.07	0.13
Respiratory, thoracic, and mediastinal disorders	4.60	1.16	-	-	0.17	0.22	0.05	0.08
Skin and subcutaneous tissue disorders	2.38	0.0	5.27	1.02	0.08	0.00	0.00	0.00
Vascular disorders	5.15	1.70	-	-	0.18	0.24	0.09	0.13
Psychiatric disorders	3.82	2.04	3.14	0.0	-	-	-	-
Blood and lymphatic system disorders	-	-	-	-	0.24	0.35	0.00	0.00
Cardiac disorders	-	-	-	-	0.17	0.22	0.09	0.13
Ear and labyrinth disorders	-	-	-	-	0.14	0.10	0.05	0.09
Endocrine disorders	-	-	-	-	0.19	0.19	0.06	0.09
Eye disorders	-	-	-	-	0.13	0.24	0.06	0.09
Hepatobiliary disorders	-	-	-	-	0.40	0.44	0.18	0.20
Neoplasms benign, malignant, and unspecified (incl cysts and polyps)	-	-	-	-	0.18	0.26	0.08	0.15
Renal and urinary disorders	-	-	-	-	0.22	0.24	0.13	0.23
Reproductive system and breast disorders	-	-	-	-	0.30	0.33	0.09	0.17

## Data Availability

All data are included in the manuscript and Supplementary Materials. Additional data and information are available upon request.

## Supplementary Materials

The following supporting information can be downloaded at https://www.mdpi.com/article/10.3390/jcm14030812/s1: Table S1: List of obesity and obesity-related disease conditions used for the search in this study. Table S2: The locations, including the countries and clinical sites, of each trial in the 10,407 clinical trials.
